# Tumour cell conditioned medium reveals greater M2 skewing of macrophages in the absence of properdin

**DOI:** 10.1002/iid3.142

**Published:** 2017-01-26

**Authors:** Izzat A.M. Al‐Rayahi, Michael J. Browning, Cordula Stover

**Affiliations:** ^1^ Department of Infection, Immunity and InflammationUniversity of LeicesterLeicesterUK; ^2^ Department of Medical Laboratory TechnologyCollege of Health and Medical TechnologyBaghdadIraq; ^3^ Department of ImmunologyLeicester Royal InfirmaryLeicesterUK

**Keywords:** Macrophages, tumour conditioned medium, complement properdin

## Abstract

**Introduction:**

The tumour microenvironment is shaped by the interaction of immune, non immune, and tumour cells present in close proximity. Tumour cells direct the development of a locally immune suppressed state, affecting the activity of anti tumour T cells and preparing the escape phase of tumour development. Macrophages in the tumour typically develop into so‐called tumour associated macrophages with a distinct profile of activities which lead to a reduction in inflammation and antigen presentation. The direct impact of tumour cell conditioned medium on the activity profile of macrophages in dependence of their complement component expression has not yet been investigated.

**Methods:**

In our in vitro study, macrophages differentiated from bone marrows of properdin deficient and wildtype mice were stimulated with conditioned medium of a syngeneic tumour cell line, B16F10, a mouse melanoma subline.

**Results:**

In comparison with macrophages from wildtype mice, those from congenic properdin deficient mice showed skewing towards M2 profile, encompassing mRNA expression for genes involved in arginine metabolism, production of type 2 cytokines, and relatively lower surface expression of molecules needed for antigen presentation.

**Conclusions:**

These data suggest that properdin insufficiency promotes a tumour environment that helps the tumour evade the immune response.

## Introduction

A component of innate immunity, complement has been characterised as a controlled, hierarchical enzymatic cascade active in the blood phase, which is activated in the presence of pathogens, immune complexes or altered self surfaces 1, 2. However, a role for complement in B‐ and T‐cell immunity has also been identified in recent years, involving engagement of complement activation products C3a and C5a with their cognate receptors, as well as membrane expression of downregulators of complement activation 2, 3. A direct cell impact of intact complement expression/activation has been described in the murine system, based on significantly altered cell activities in genetically targeted complement deficient mice 4. Indeed, as more details become known on the intense crosstalk between complement and other cell effector mechanisms, complement knockouts are likely to reveal an importance other than the defect in the humoral activation cascade or its regulation, namely on cellular activities more generally 5. This is the background against which current research revisits proposals from the 1970s of an involvement of complement activation in tumour immunity 6, 7. Our understanding of the role of complement in tumour growth, the anti tumour immune response and in treatment of tumours has been extensively reviewed 8.

Properdin is a positive regulator of complement activation, by stabilising inherently labile enzyme complexes thereby enhancing complement activity 1. The properdin deficient mouse line used in this study was generated in order to investigate the role of properdin in disease models of infection and inflammation, hypothesising that the significance of properdin for an organism exceeded its protective role in immunity towards *Neisseria menigitidis* (certain serotypes) 9.

Previous work using experimental models of monomicrobial sepsis suggested greater M2‐type cell skewing in properdin deficient mice on C57Bl/6J background compared to their congenic wildtype controls 10. Others have shown, using bone marrow derived macrophages from C57Bl/6J, that conditioned medium from syngeneic lung carcinoma cells could influence in vitro the profile of macrophages towards M2 phenotype, typical of tumour‐associated macrophages observed in vivo 11.

Typically in mouse, macrophages of a so‐called M1 phenotype are characterised by an up‐regulation of inducible nitric oxide synthase (iNOS) that generates nitric oxide from L‐arginine 12 and by the producton of IL‐1β,TNFα, IL‐12, IL‐23, CXCL9 and CXCL10 13. On the other hand, macrophages of a so‐called M2 phenotype produce high levels of arginase‐1, IL‐10 and IL‐1RA. In a non orthotopic syngeneic tumour model, arginase 1 activity was significantly correlated with tumour volumes (reaching up to 4 ml) in wildtype mice 14. M2 macrophages promote tumour progression by stimulating angiogenesis, matrix remodelling and inhibition of adaptive immune response 15.

The aim of this study was to evaluate in vitro the extent to which syngeneic tumour cell conditioned medium would exert an effect on skewing cell phenotypes and functions relevant to tumour growth, and whether macrophages derived from properdin deficient and wildtype mice differed in their reactions. B16F10 is a well characterised subline of C57BL/6J melanoma 16, 17 and was used as a tumour cell line to generate conditioned medium. We pursued the hypothesis that macrophages express an activity profile towards tumour cell conditioned medium that was modulated by complement properdin.

## Methods

### Preparation of B16F10 tumour cell conditioned medium

B16F10 cells, a pigmented mouse melanoma cell line kindly provided by Professor Stephen Todryk (Northumbria University, Newcastle upon Tyne, UK), were cultured in DMEM supplemented with 10% (v/v) fetal calf serum, 2 mM l‐glutamine, 100 U/ml penicillin, 100 µg/ml streptomycin. When cells reached about 70% confluence, the medium was collected and centrifuged at 350 g. The supernatant was filtered (0.2 μm) to serve as the conditioned medium and was used fresh.

### Mice

Age and sex matched congenic mice were taken from the properdin deficient mouse colony held at University of Leicester after genotyping 18. Mice were housed in groups in ventilated cages at 21°C, 50% humidity, with 12/12 h light/dark cycle, and had *ad libitum* access to food and water. They were humanely killed by cervical dislocation and exsanguination by competent animal technicians. All regulated procedures were in accordance with UK Home Office regulations and had received institutional ethical approval.

### Differentiation of macrophages from mouse bone marrows

Macrophages were prepared from femur and tibia bones from matched genotypes as previously described 10, but a recent guideline paper was followed for the differentiation step 19. After erythrocyte lysis, three experimental groups were set up, each using a concentration of 1 × 10^6^ cells/ml: Cells were pretreated with 20 ng/ml GM‐CSF (Granulocyte/Macrophage Colony‐Stimulating Factor; PeproTech EC Ltd., London, UK) for 7 days, during which time they typically developed an elongated shape 20 and 95% of the population was CD11b positive (data not shown). Adherent cells were stimulated for 24 h with LPS *Escherichia coli* 0111:B4 (100 ng/ml; Invivogen, Toulouse, France) + IFNγ (20 ng/ml, eBioscience, San Diego, CA), or B16F10 conditioned medium, or left unstimulated. The supernatant was collected from each well and aliquoted in reaction tubes, then stored at −80°C for quantification of IL‐12 and IL‐10.

### RNA extraction and quantitative real‐time PCR

Total RNA was extracted from cells stimulated with LPS and IFNγ or B16F10 conditioned medium using RNeasy Mini Kit (Qiagen). Contaminating genomic DNA was removed from RNA samples using the RNase‐Free DNase Set (Qiagen). 2 µg total RNA was reverse‐transcribed into cDNA using first strand synthesis kit (Thermoscientific). Gene‐specific amplification using SensiMix SYBR kit (Bioline Reagents Ltd., London) was analysed with Rotor‐Gene 6000 (Corbett Life Science) for IL‐6, IL‐1β, iNOS, TNF‐α, IL‐10, arginase‐1 and MCP‐1 in comparison with GAPDH. Sequences of priming oligonucleotides are given in Table 1. ΔΔCT values were used 21, the mRNA expression corrected for GAPDH and compared with unstimulated BMDM. Amplifications were set up in duplicates.

**Table 1:** Sequences of oligonucleotide primers used in this study

Primers, mouse	Sequence	Size (bp)	T_A_ (°C)	Reference sequence accession number NCBI
GAPDH	5′‐CCTGGAGAAACCTGCCAAGTATG‐3′	132	55	NM_0080848
	5′‐AGAGTGGGAGTTGCTGTTGAAGTC‐3′			
IL‐10	5′‐CCCTGGGTGAGAAGCTGAAG‐3′	84	58	NM_010548
	5′‐CACTGCCTTGCTCTTATTTTCACA‐3′			
TNF‐α	5′‐GGCAGGTCTACTTTGGAGTCATTGC‐3′	333	55	NM_0013693
	5′‐ACATTCGAGGCTCCAGTGAATTCGG‐3′			
iNOS	5′‐TAAAGATAATGGTGAGGGG‐3′	270	60	NM_010927
	5′‐GTGCTTCAGTCAGGAGGTT‐3′			
Arginase‐1	5′‐AGGAACTGGCTGAAGTGGT‐3′	220	60	NM_007482
	5′‐GATGAGAAAGGAAAGTGGC‐3′			
IL‐6	5′‐GACAACTTTGGCATTGTGC‐3′	160	53	NM_031168.1
	5′‐ATGCAGGGATGATGTTCTG‐3′			
MCP‐1	5′‐ CACTCACCTGCTGCTACTCATTCAC‐3′	490	57	NM_0011333
	5′‐GGATTCACAGAGAGCGAAAAATGG‐3′			
IL‐1β	5′‐TTGACGGACCCCAAAAGATG‐3′	200	55	NM_008361
	5′‐ AGAAGGTGCTCATGTCCTCA‐3′			

### ELISA

Sandwich ELISAs were purchased from Peprotech for quantification of murine IL‐12 and IL‐10 using undiluted serum samples. Standard curves were plotted and levels corresponding to absorbances (represented by the standard curve) were calculated using GraphPad prism.

### Flow cytometry

GM‐CSF differentiated macrophages, unstimulated and stimulated with B16F10 conditioned medium, were used. Single cell suspensions were prepared by treating 2.5 × 10^6^–3 × 10^6^ cells with trypsin followed by gentle scraping with a cell scraper. Individual cell preparations were stained for cell surface markers using either one or a combination of fluorochrome conjugated monoclonal antibodies with their isotype controls (Table 2). FACS buffer (PBS supplemented with 3% (v/v) FCS) was used throughout the procedure for preparation, as wash buffer and for the dilution of the antibodies. Cells were stained in 96‐well round bottom microtiter plates. 100 μl cell suspension from each sample were stained with 50 μl purified anti‐Fc receptor blocking antibody (anti‐CD16/CD32 from Biolegend) diluted 1:200. After incubation on ice for 30 min, cells were washed and stained by adding 50 μl/well of the appropriate antibody or antibodies on ice in the dark for 30 min. The plate was centrifuged (300 g, 5 min). Cells were washed and resuspended in a total volume of 400 μl PBS‐3% (v/v) FCS. Spectral overlap of fluorochromes was compensated where necessary. Flow cytometry data was acquired using Canto II (BD) and analysed using FlowJo software (version 8.8.3, Tree Star).

**Table 2:** Antibodies used in this study

Ab	Fluorophore	Clone	Dilution	Company
CD80 (B7‐1)	FITC	16‐10A1	1:250	BD Bioscience
CD86(B7‐2)	FITC	GL‐1	1:250	BD Bioscience
MHC‐II (I‐A/I‐E)	FITC	2G9	1:250	BD Bioscience
CD11b	PE	M1/70	1:300	Biolegend, ebioscience
CD206	APC	C068C2	1:250	Biolegend
IL‐12/IL‐23	APC	C15.6	1:250	Biolegend

To perform intracellular staining, 96 well round bottom plates were used. Cellular secretion of cytokines was blocked by incubating the cells (2–5 × 10^5^ cells per well) in RPMI medium supplemented with Brefeldin (3 μg/ml, eBiosciences). Cytokine production was induced by adding phorbol 12‐myristate 13‐acetate (PMA) (50 ng/ml) and ionomycin (500 ng/ml). After 6 h at 37°C, the cells were washed and stained with antibodies against extracellular markers as described previously. Fix/Perm solution (100 μl of 1:4 dilution; BD Biosciences) was added and the cells were further incubated for 20 min. Then the cells were washed with 100 μl Perm/Wash buffer (1:10 diluted in nanopure water) (BD Biosciences). Subsequently staining with antibodies against intracellular IL‐12 (Table 2), diluted in Perm/Wash buffer, was carried out. After washing, cells were resuspended in 400 μl PBS‐3% (v/v) FCS. For this analysis, macrophages from three mice of each genotype were pooled.

### Statistical analyses

Statistical analysis was performed using GraphPad v6.0 (GraphPad Software) using two‐way ANOVA (with Bonferroni's multiple comparisons) or unpaired one‐tailed Mann–Whitney *U* tests (comparisons between two groups). *P*‐values less than 0.05 were considered significant.

## Results

Culturing mouse bone marrow cells in the presence of GM‐CSF leads to differentiation of adherent macrophages 22 which can be followed for M1 or M2 skewing on stimulation. Conditioned medium obtained from mouse melanoma subline B16F10 was used to stimulate BMDM from wildtype and properdin deficient mice to investigate whether this stimulation would skew cells towards M1 or M2 phenotype and whether the different genotypes modulated their reactivities to this stimulus. Quantitative RT‐PCR, ELISA and FACS were used to measure changes in cytokine production and phenotypic markers associated with macrophage polarisation. For the purpose of this work, the function‐driven categorisation of M1 and M2 put foward by Ch Mills is followed 23.

### Analysis of B16F10‐mediated skewing of BMDM by qPCR

**Figure 1:**
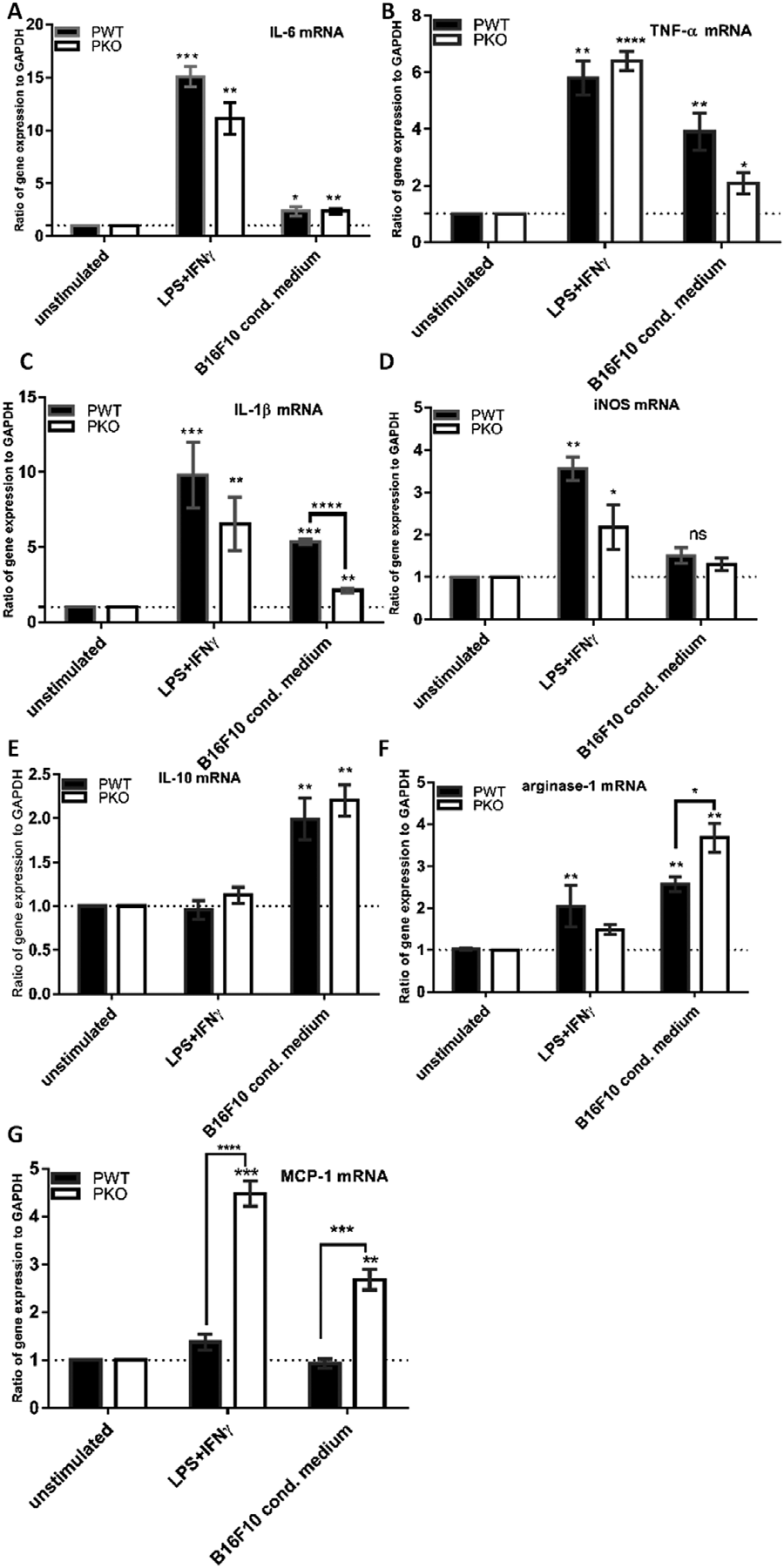
Analysis of mRNA expression for characteristic markers of M1/M2 macrophages skewing in naïve mice. BMDM from wildtype (*n* = 4) and properdin‐deficient mice (*n* = 5) were treated with GM‐CSF (20 ng/ml) for 7 days and stimulated with LPS (100 ng/ml) + IFNγ (20 ng/mL) or with B16F10 conditoned medium. cDNA was prepared to quantify gene expression of IL‐6, IL‐1β, iNOS, TNF‐α, IL‐10, arginase‐1 and MCP‐1 in comparison with GAPDH. ΔΔCT value were used, the mRNA expression corrected for GAPDH and compared in relation to unstimulated BMDM. The qPCR results represent at least three experiments, each set up in duplicate. The data are presented as means ± SEM. Statistical analyses were performed using two‐way ANOVA with Tukey's multiple comparisons test (significances on top of columns compare to the respective unstimulated controls) and one‐tailed Mann–Whitney *U* test (significances with brackets).

IL‐6, TNF‐α, IL‐1β and iNOS were used as indicators of M1 phenotype. There was a robust stimulation of IL‐6, TNF‐α and IL‐1β mRNA expression in BMDM from both genotypes stimulated with LPS and IFNγ compared to unstimulated cells (Fig. 1A–C). Incubation of BMDM with B16F10 conditioned medium led to a significant increase of IL‐6, TNF‐α and IL‐1β mRNA by both genotypes compared to unstimulated cells (Fig. 1A–C). However, BMDM from properdin deficient mice when exposed to B16F10 conditioned medium produced significantly less IL‐1β mRNA compared to BMDM from wildtype mice (Fig. 1C). iNOS mRNA was induced only in BMDM exposed to classic stimulators of M1 activity (LPS and IFNγ) (Fig. 1D). There was no appreciable increase of iNOS mRNA after incubation with B16F10 conditioned medium in either genotype compared to unstimulated BMDM (Fig. 1D).

IL‐10, arginase and MCP‐1 were used as indicators of M2 phenotype. There was a robust induction of IL‐10 mRNA in BMDM from wildtype and properdin deficient mice exposed to B16F10 conditioned medium (Fig. 1E). Arginase‐1 mRNA and MCP‐1 mRNA expressions after stimulation with B16F10 conditioned medium were discriminatory for BMDM between wildtype and properdin deficient mice: BMDM from properdin deficient mice increased their arginase‐1 mRNA expression to a significantly greater extent than BMDM from wildtype mice when exposed to B16F10 conditioned medium (Fig. 1F), while LPS and IFNγ increased arginase‐1 mRNA expression in BMDM from wildtype, but not from properdin deficient mice, compared to unstimulated BMDM. In the analysis of MCP‐1 mRNA, we found that BMDM from properdin deficient mice were significantly more sensitive to upregulate their MCP‐1 mRNA expression in response to LPS and IFNγ as well as to B16F10 conditioned medium, while BMDM from wildtype mice showed no change of their mRNA for MCP‐1 under either of these conditions (Fig. 1G).

Taking together the reactivities recorded in BMDM from wildtype mice stimulated with B16F10 conditioned medium in relation to mRNA expressions of unstimulated cells, B16F10 conditioned medium led to a significant increase of mRNA expression for IL‐6, TNF‐α, IL‐1β, IL‐10, and arginase‐1. For the genes tested, BMDM from properdin deficient mice had a reproducibly altered response to B16F10 conditioned medium for IL‐1β and arginase‐1 mRNA (Fig. 1C, F), and a consistently altered response to both LPS and IFNγ and B16F10 conditioned medium for MCP‐1 mRNA (Fig. 1G): In response to B16F10 conditioned medium, BMDM from properdin deficient mice had significantly lower levels of IL‐1β mRNA and higher levels of arginase‐1 and MCP‐1 mRNA than BMDM from wildtype mice. BMDM from properdin deficient mice therefore displayed M2 like responses for arginase‐1 and MCP‐1 mRNA, compared with BMDM from wildtype mice, which gave a stronger IL‐1β (M1) response to B16F10 conditioned medium.

### Analysis of B16F10‐mediated skewing of BMDM by cytokine ELISA

**Figure 2:**
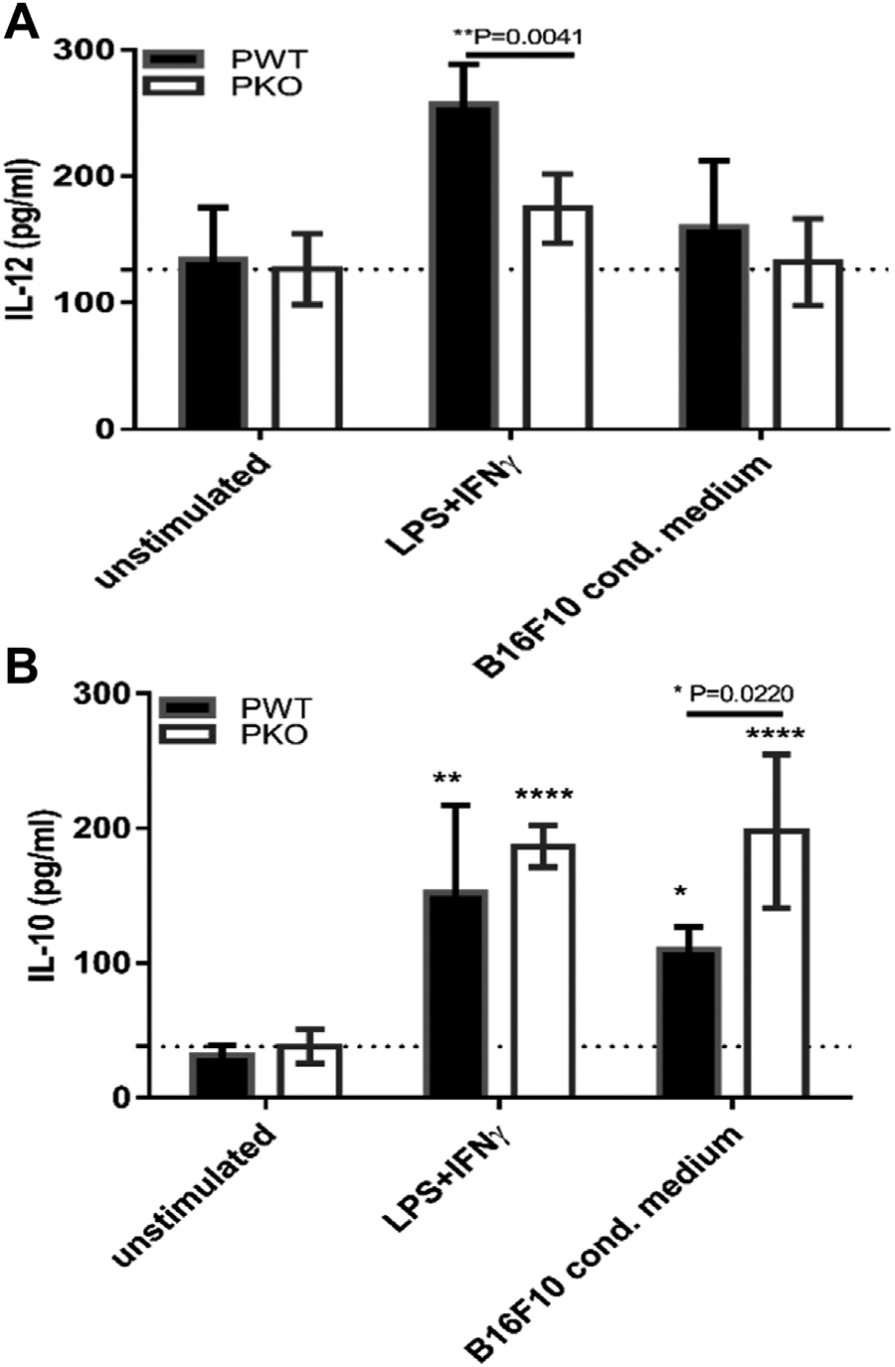
Levels of IL‐12 and IL‐10 in supernatants of GM‐CSF differentiated BMDM after stimulation. BMDM from wildtype (*n* = 4) and properdin‐deficient (*n* = 5) mice were treated with GM‐CSF (20 ng/ml) for 7 days and stimulated with LPS (100 ng/ml) + INF‐ɣ (20 ng/ml) or B16F10 conditioned medium. Unstimulated cells were used as control. Supernatants were used for measurement of cytokines (A, IL‐12; B, IL‐10) by ELISA. Results represent at least four experiments each set up in duplicate. The data are presented as means ± SEM. Statistical analyses were performed using two‐way ANOVA with Tukey's multiple comparisons test (significances on top of columns compare to the respective unstimulated controls) and one‐tailed Mann–Whitney *U* test (significances with brackets).

IL‐12 and IL‐10 represent characteristic cytokines in types 1 and 2 cellular immune responses, respectively. Because complement activity, its effector mechanisms and levels of IL‐12 interact 24, IL‐12 was measured in supernatants obtained from those cultures which were analysed for their mRNA expressions (see above). BMDM from properdin deficient mice were significantly impaired in their LPS and IFNγ induced release of IL‐12 compared to BMDM from wildtype mice (Fig. 2A). Conversely, IL‐10 release was significantly greater in the supernatant of BMDM from properdin deficient mice than wildtype mice when exposed to B16F10 conditioned medium (Fig. 2B).

### Analysis of B16F10‐mediated skewing of BMDM by flow cytometry

**Figure 3:**
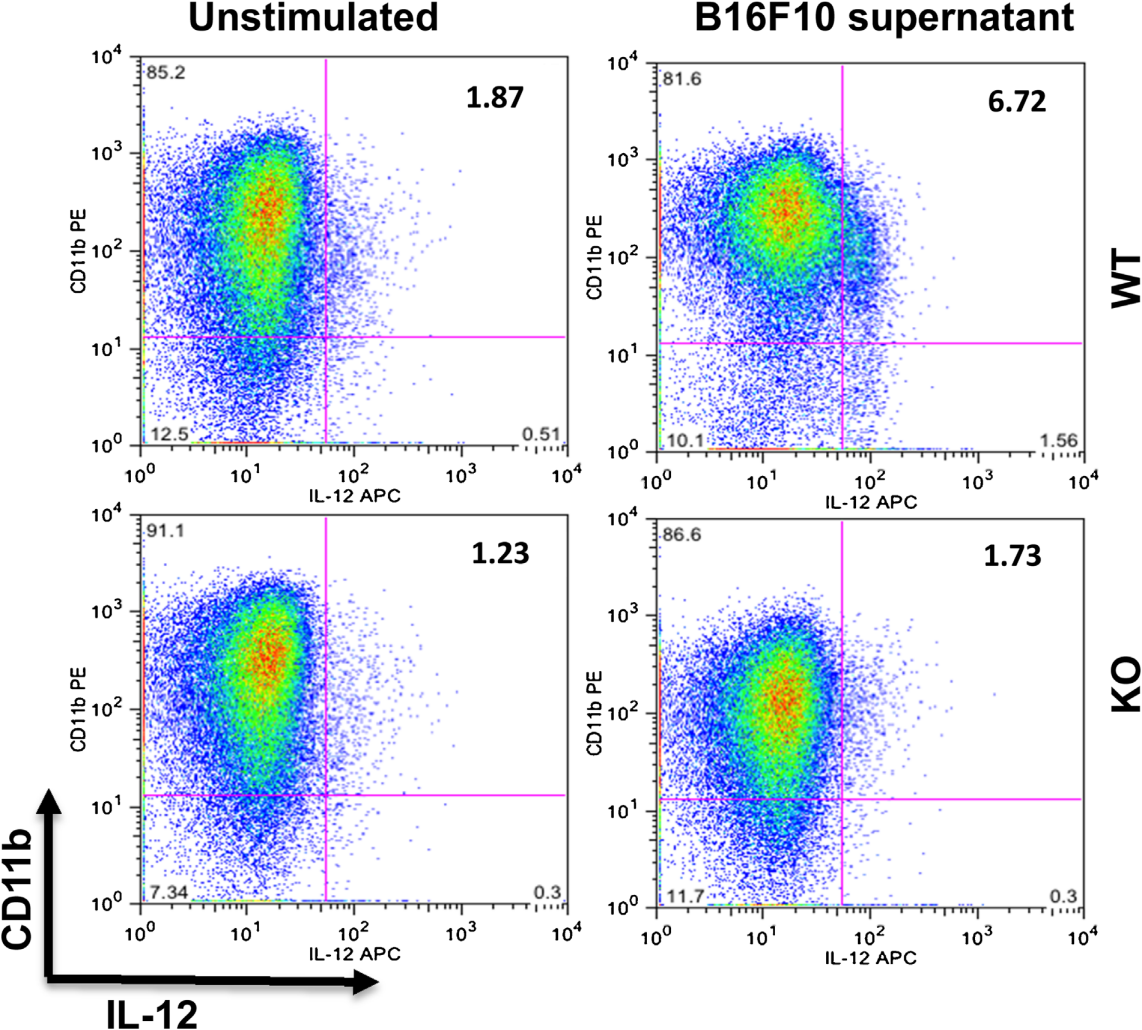
Intracellular staining for IL‐12 of CD11b^+^ BMDM stimulated with B16F10 conditioned medium. BMDM were pooled from wildtype (*n* = 3) and properdin‐deficient (*n* = 3) mice and treated with GM‐CSF (20 ng/ml) for 7 days. Then the cells were stimulated with B16F10 supernatant. Unstimulated cells were used as a control. Samples were stained for the expression of CD11b and intracellular IL‐12.

In order to identify the numbers of macrophages producing Il‐12, intracellular staining for IL‐12 was performed in CD11b^+^ cells. This analysis revealed that the prevalence of CD11b^+^IL‐12^+^ was increased three to fourfold in BMDM from wildtype mice stimulated with B16F10 supernatant, in contrast with BMDM from properdin deficient mice, which showed no increase (Fig. 3). These results further support a skewing to M2 phenotype in BMDM from properdin deficient mice exposed to B16F10 conditioned medium, compared with wildtype.

**Figure 4:**
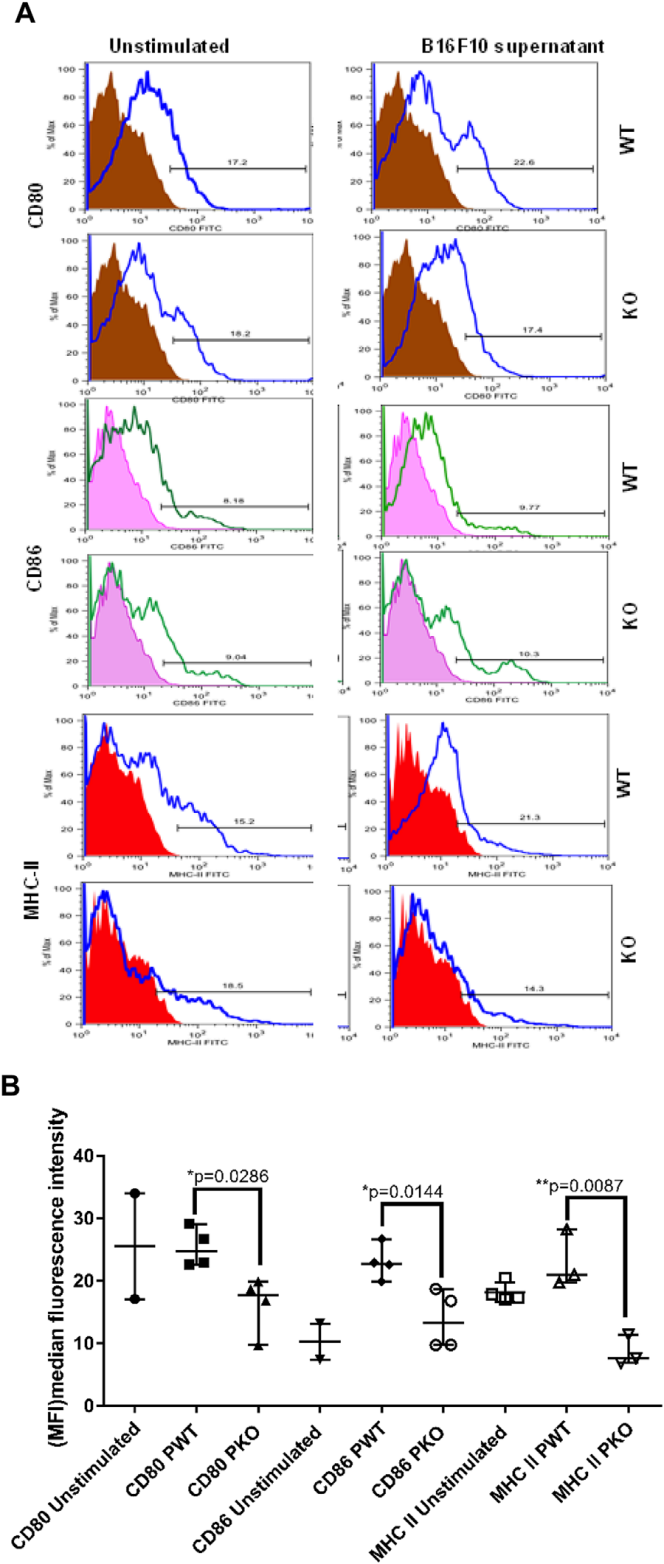
Surface receptor expression (CD80, CD86 and MHC II) of GM‐CSF differentiated BMDM after stimulation with B16F10 conditioned medium. BMDM from properdin deficient and wildtype mice were differentiated with GM‐CSF for seven days and then stimulated B16F10 conditioned medium for 24 h. Unstimulated cells were used as control. Example histograms of antibody staining (specific and isotype control) are shown in A, cumulative analysis of (MFI) median fluorescence intensity is shown in B. The data are presented as means ± SEM. Statistical analysis was performed by one‐tailed Mann–Whitney *U* test (*P < 0.05).

Next, we examined the effect of B16F10 conditioned medium on the expression of MHCII, CD80 and CD86 in BMDM from properdin deficient and wildtype mice. MHC II is expressed on antigen presenting cells such as macrophages, and CD80 and CD86 are important T cell co‐stimulatory molecules. The expression of these molecules was used as an indication of pro‐inflammatory, M1‐type macrophages. Stimulation with B16F10 conditioned medium was associated with upregulation of MHC II, CD80 and CD86 in pooled samples of BMDM from wildtype mice that was not seen in BMDM from properdin deficient mice (Fig. 4A, B).

**Figure 5:**
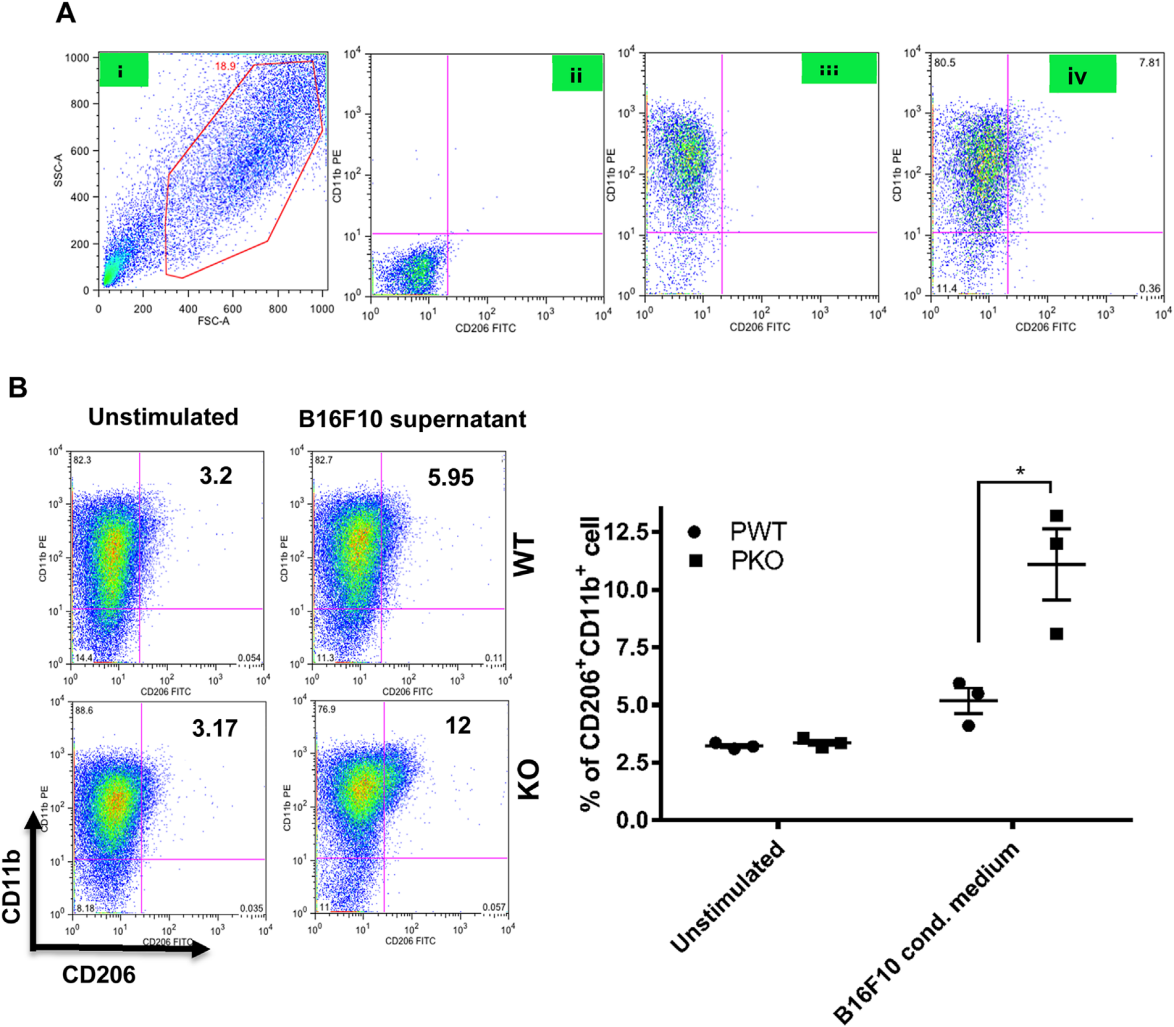
Expression of CD206 and CD11b in GM‐CSF differentiated BMDM cells after stimulation with B16F10 conditioned medium. A: (i) Strategy of CD11b+CD206+ cell gating on the main macrophage population; (ii) unstained cells; (iii) single stain for only CD11b+ cell labelled with PE flurochrome; (iv) cells stained with both CD11b and CD206. B: BMDM from wildtype and properdin‐deficient mice were differentiated with GM‐CSF (20 ng/ml) for 7 days and stimulated with B16F10 conditioned medium. Unstimulated cells were used as controls. Cells were stained with CD206 FITC and CD11b PE. Percentage of double positive (CD11b+CD206 +) was calculated by using Flow Jo software. Results represent three independent experiments. The data are presented as means ±SEM. Statistical analysis was performed by one‐tailed Mann–Whitney *U* test (*P < 0.05).

Finally, a CD206^+^CD11b^+^ double positive phenotype was used as an indication of M2 polarised macrophages. Stimulation with B16F10 conditioned medium resulted in a significantly higher percentage of CD206^+^CD11b^+^cells in BMDM from properdin deficient mice when compared to wildtype. There was a significant induction of this phenotype by B16F10 conditioned medium in BMDM from properdin deficient mice compared with unstimulated cells (*P* = 0.0391) (Fig. 5), whereas B16F10 conditioned medium did not significantly increase the percentage of CD206^+^CD11b^+^cells in BMDM from wildtype mice.

## Discussion

Tumour environments are complex milieus to dissect analytically. This study used GM‐CSF responsive, adherent primary cells from bone marrows of mice as an in vitro model to investigate the influence of soluble products from an established C57Bl/6J melanoma cell line (contained in the conditioned medium) on macrophage phenotypes, and assess a modulatory role of complement properdin in cell reactivities. This in vitro approach differs from previous studies investigating the role of complement in shaping the antitumour response using ex vivo and in vivo experiments 14.

Upon stimulation, macrophage activation is directed towards either classic, M1, or alternative, M2, phenotype. M1 macrophage activation depends on Toll‐like receptors (TLRs) and activation of nuclear factor kappa B/c‐Jun N‐terminal kinase 1, leading to the production of the inflammatory cytokines and of inducible nitric oxide synthase, iNOS. On the other hand, M2 macrophage activation depends on engagement of peroxisome proliferator‐activated receptors (PPARs) or IL‐4‐STAT6 pathways and results in an anti‐inflammatory response that is accompanied by upregulation of mannose receptor CD206, and arginase‐1. M1 and M2 cells are maximally skewed cell phenotypes within a continuum of macrophage activation and are preferentially expanded in different inflammatory contexts 25.

In this study, B16F10 conditioned medium provoked M2‐type behaviour in BMDM, as demonstrated by the lack of an increase in iNOS mRNA, and by a two‐fold induction of IL‐10 mRNA. IL‐10 secretion into the supernatant after 24 h stimulation with B16F10 conditioned medium was significantly greater from BMDM differentiated from properdin deficient mice compared to wildtype mice, pointing to different posttranscriptional regulation of IL‐10 mRNA by the genotypes. These results cannot be explained by the secretion of IL‐10 by B16F10 cells, as IL‐10 levels in B16F10 conditioned medium were below the detection limit (data not shown).

Studies on in vitro cultured cancer cells have led to the emergence of concepts which link M1 cells with increased killing of tumour cells, while M2 cells were correlated with protumoural activity. Recently, an elevated M1/M2 ratio was associated with favourable prognosis in ovarian cancer. Quantification of the M1/M2 ratio in both tumour and stroma revealed that only the M1/M2 ratio of macrophages present intratumorally were prognostic 26.

This study shows for the first time that B16F10 conditioned medium enhanced the expressions of arginase‐1 and MCP‐1 mRNA significantly in BMDM from properdin deficient compared to wildtype mice. The increase of MCP‐1 mRNA with a concomitant decrease of Il‐1β mRNA by BMDM from properdin deficient compared to wildtype mice when exposed to B16F10 conditioned medium is suggestive of a profile of expression associated with increased tumour growth 27, 28.

IL‐12 is centrally important to the instruction of antitumour T cells 29. While BMDM from properdin deficient mice did not show any accumulation of intracellular IL‐12 after stimulation with B16F10 conditioned medium, BMDM from wildtype mice had increased the presence of intracellular IL‐12, although the secretion of IL‐12 into the supernatant, as assessed by ELISA in a parallel experiment, did not reveal a significant difference between the B16F10 conditioned medium treated BMDM of both genotypes. The fact that BMDM from wildtype mice were able to respond with an increase of IL‐12 secretion is shown when they are exposed to LPS and IFNγ for 24 h, producing significantly elevated levels in the supernatant compared to LPS and IFNγ stimulated BMDM from properdin deficient mice. Therefore, the IL‐12 amounts elicited by the action of soluble tumour cell products (as captured by sensitive intracellular staining using FACS) may be sufficient in a microenvironmental context, where complement products are proposed to be active 30.

In contrast, BMDM from properdin deficient mice, in the presence of B16F10 conditioned medium released significantly elevated levels of IL‐10 into the supernatant, when compared with BMDM from wildtype mice. In addition, BMDM from properdin deficient mice were significantly impaired in their surface expression of MHC‐II, CD80 and CD86 compared to wildtype following exposure to B16F10 conditioned medium, which is consistent with the expression of M2 macrophage phenotype 31, 32. The possibility that BMDM from properdin deficient mice were simply immature rather than differently polarised, can be counterargued by the simultaneous increase of cell surface staining for CD206, a marker indicative of M2 macrophages 33.

The above in vitro findings demonstrate a significantly altered phenotype of BMDM from properdin deficient mice in response to tumour cell conditioned medium compared to wildtype mice (skewed towards high IL‐10 protein, high MCP‐1 mRNA, high arginase‐1 mRNA, low Il‐1β mRNA, M2 surface marker profile). Following this study of relative replacement, only in vivo experimentation will demonstrate the relevance of a macrophage phenotype in the absence of properdin with strong features of tumour associated macrophage population for tumour growth and development, as well as for the shaping of the tumour microenvironment. A greater need for mouse orthotopic cancer models to investigate the role of complement in tumour has recently been raised 34. Indeed, more relevant, compartment specific data are required to add to the analyses of the importance of C5a in tumour models, as studies so far yield opposing conclusions 14, 35 and of the importance of MCP‐1 in complement deficient systems. Further in vitro studies will define the differences in intracellular signalling of macrophages differentiated from properdin deficient and wildtype mice that yield a distinct inflammatory response to melanoma cell conditioned medium.

## Conflict of Interest

None declared.
